# Volatile Profile, Phytochemicals and Antioxidant Activity of Virgin Olive Oils from Croatian Autochthonous Varieties *Mašnjača* and *Krvavica* in Comparison with Italian Variety *Leccino*

**DOI:** 10.3390/molecules19010881

**Published:** 2014-01-14

**Authors:** Mladenka Šarolić, Mirko Gugić, Carlo Ignazio Giovanni Tuberoso, Igor Jerković, Marko Šuste, Zvonimir Marijanović, Piotr Marek Kuś

**Affiliations:** 1 Department of Food Technology, Marko Marulić Polytechnic in Knin, Petra Krešimira IV, 30, Knin 22300, Croatia; E-Mails: msarolic@veleknin.hr (M.S.); mgugic@veleknin.hr (M.G.); msuste@veleknin.hr (M.S.); zmarijanovic@veleknin.hr (Z.M.); 2 Department of Life and Environmental Sciences, University of Cagliari, via Ospedale 72, Cagliari 09124, Italy; E-Mail: tuberoso@unica.it; 3 Department of Organic Chemistry, Faculty of Chemistry and Technology, University of Split, N. Tesle 10/V, Split 21000, Croatia; 4 Department of Pharmacognosy, Wrocław Medical University, ul. Borowska 211a, Wrocław 50-556, Poland; E-Mail: kus.piotrek@gmail.com

**Keywords:** *Mašnjača*, *Krvavica*, *Leccino*, virgin olive oil, volatile compounds, phenols, antioxidant activity (DPPH)

## Abstract

Virgin olive oils (VOOs) obtained from the fruits of Croatian autochthonous varieties *Mašnjača* and *Krvavica* were extensively characterized for the first time. Investigated oils were compared with the oil obtained from Italian variety *Leccino*, grown and processed under the same conditions. Headspace volatile profile, tocopherols, chlorophylls, carotenoids and total phenolic content, peroxide value, % acidity, K_232_, K_270_ as well as antioxidant activity (DPPH) of the oils’ hydrophilic fractions (HFs) including their phenolic composition were assessed by means of HS-SPME/GC-MS, HPLC-FL, HPLC-DAD and spectrophotometric methods, respectively. Most of the studied quality parameters varied between the cultivars. The main volatile compounds detected in all tested olive oils were the C6 compounds derived from polyunsaturated fatty acids through the lipoxygenase pathway. *Krvavica* oil was characterized by hexanal (8.8%–9.4%). *Leccino* oil contained the highest percentage of (*E*)-hex-2-enal (73.4%–74.0%), whereas (*Z*)-hex-3-enal (21.9%–25.0%) and (*E*)-hex-2-enal (27.6%–28.9%) dominated in *Mašnjača* oil. *Leccino* oil contained the highest amount of tocopherols (312.4 mg/kg), chlorophylls (7.3 mg/kg), carotenoids (4.2 mg/kg) and total phenols (246.6 mg/kg). The HF of *Leccino* oil showed the highest antioxidant capacity (1.3 mmol TEAC/kg), while the HFs of *Mašnjača* and *Krvavica* oils exhibited the activity of 0.5 mmol TEAC/kg.

## 1. Introduction

Virgin olive oil (VOO) exhibits nutritional and sensory characteristics that make it valuable component of Mediterranean diet. It is produced in large quantities from different varieties of *Olea europaea* L. fruits using physical methods. VOO shows sensory characteristics and nutritional properties that distinguish it from the other oil types. VOOs obtained from fresh and undamaged (neither mechanically nor by insects and moulds) olive fruits, after proper processing, are characterized by a delicate and unique flavour highly appreciated by consumers. Their peculiar attractive taste and aroma is related to several non-volatile compounds and to a number of volatile organic compounds (VOCs) [1]. Most of the VOCs are formed during the mechanical extraction process through the action of enzymes that are released when the fruits are crushed, and continue to form during malaxation in the enzymatic reactions known as lipoxygenase pathway [2]. The VOCs of virgin olive oil depends on several factors, such as the level and activity of the enzymes involved in various pathways [3,4], the cultivar [5,6], the ripening cycle of the fruit [7], the processing equipment [8], the extraction method and storage conditions [9], the climate and soil type [10] and the growing area [11]. VOOs have been particularly appreciated due to high stability with respect to other vegetable oils, which may be caused by the presence of phenolic compounds and tocopherols.

Due to the high content of beneficial constituents, such as monounsaturated fatty acids (MUFAs) and phenolic compounds, olive oil consumption has been steadily increasing all over the world and drives olive cultivation in new areas such as Latin America, California, South Africa, China, and Australia [12]. According to some authors, the beneficial impact of olive oil on health is mainly attributed to its richness in monounsaturated and low unsaturated fatty acids as well as to polyphenols (which act as natural antioxidants) that may together contribute to the prevention of several human diseases such as coronary heart disease, cognitive impairment, colon cancer and many others [13]. The nutritional value of VOO arises not only from high levels of oleic acid and the presence of hydrophilic phenols but also from minor components such as phytosterols, carotenoids and tocopherols [14]. The minor components present in the olive oil were the subject of many studies [15,16]. Phenol types and concentrations differ greatly, since many factors such as cultivars [17], ripening degree, seasonal variation, climate and area of origin [10] affect phenolic content in the olive oil. VOOs can be also considered as an example of a functional food containing a variety of components that may contribute to their overall therapeutic characteristics. 

Lately, special attention has been focused on researching monovarietal oils and the oils containing various forms of protection (e.g., geographical, ecological, *etc.*), which significantly contributes to the popularization of olive oil and strengthening of the economy through the olive sector. In this study, the large spectrum of quality parameters of VOOs obtained from Croatian autochthonous varieties *Mašnjača* and *Krvavica* were analysed, to the best of our knowledge, for the first time. The aim of this work was: (1) to investigate the volatile profile and content of several non-volatile (especially those biologically active) compounds; as well as to (2) assess antioxidant activity of hydrophilic oil fractions in DPPH test; and (3) to compare the characteristic of *Mašnjača* and *Krvavica* oils with the oil obtained from the popular Italian variety *Leccino*. The *Leccino* variety has been used as the reference since it is established olive variety in the major olive growing countries and it is the most present foreign variety in Croatia.

## 2. Results and Discussion

### 2.1. Basic Quality Parameters of Investigated Virgin Olive Oils

The basic quality parameters such as acidity, peroxide value (PV), K_232_ and K_270_ values of the investigated VOOs were assessed according to EU method and the results are presented in Table 1. 

**Table 1:** Mean values of basic quality indices (% acidity, peroxide value, K_232_, K_270_) of *Mašnjača*, *Krvavica* and *Leccino* VOOs.

No.	Cultivar	Acidity ^a^ (%)	Peroxide value ^b^ (mEq O_2_/kg)	K_232_^c^	K_270_^d^
1.	*Krvavica*	0.22 ± 0.01	3.35 ± 0.12	1.62 ± 0.1	0.12 ± 0.02
2.	*Mašnjača*	0.14 ± 0.02	3.32 ± 0.16	1.58 ± 0.4	0.11 ± 0.01
3.	*Leccino*	0.15 ± 0.01	5.37 ± 0.10	1.65 ± 0.8	0.14 ± 0.02

All values are expressed as mean of triplicate determinations ± SD; ^a^ threshold value for extra VOO is ≤0.8; ^b^ threshold value for extra VOO is ≤20; ^c^ threshold value for extra VOO is ≤2.5; ^d^ threshold value for extra VOO is ≤0.22.

According to the International Olive Oil Council, acidity should not exceed 0.8% for extra virgin olive oils (IOC/T.15/NC n° 3/Rev.7, 2012 [18]). The percentual acidity for the olive oils tested ranged from 0.14% to 0.22%. *Krvavica* oil showed the highest acidity, while *Mašnjača* oil showed the lowest acidity. Peroxide value is a measure of the primary products of auto-oxidation (hydroperoxides) of an olive oil and should not exceed 20 mEq O_2_/kg for extra VOO. PV for olive oils tested ranged from 3.32 to 5.37 mEq O_2_/kg. *Mašnjača* oil showed the lowest PV (3.32 mEq O_2_/kg), while *Leccino* oil showed the highest PV (5.37 mEq O_2_/kg). K_232_ is a measure of conjugated dienes and their oxidation products that absorb at λ = 232 nm and K_270_ is a measure of conjugated trienes and secondary oxidation products (carbonyl compounds) which absorb at λ = 270 nm. K_232_ and K_270_ should not exceed 2.50 and 0.22, respectively for extra VOO. K_232_ and K_270_ values for the olive oils tested ranged from 1.58 to 1.65 and from 0.11 to 0.14 respectively. *Mašnjača* oil showed the lowest K_232_ and K_270_ value while *Leccino* oil showed the highest K_232_ and K_270_ values. The parameters for all studied olive oils (Table 1) were within estimated limits of EC Reg. 1989/2003 (2003) [19], which place them in the category of extra VOOs.

### 2.2. Volatile Compounds

**Scheme 1:**
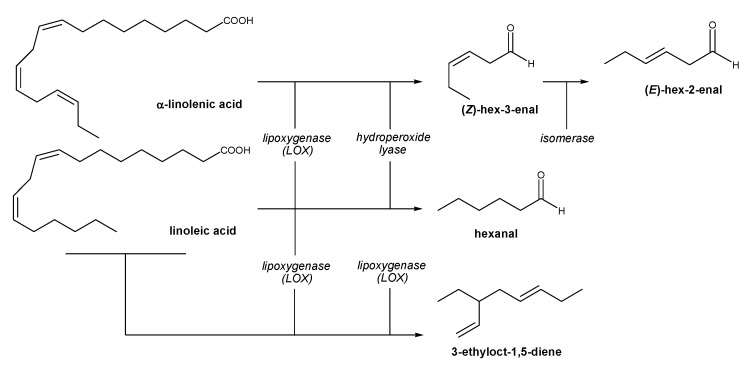
Biosynthesis of major VOCs by the lipoxygenase pathway.

HS-SPME coupled to GC-MS was utilized to characterize the VOCs in *Mašnjača*, *Krvavica* and *Leccino* VOOs. Identified C_6_ VOCs in investigated olive oils were mainly aldehydes such as hexanal, (*E*)-hex-2-enal and (*Z*)-hex-3-enal (Table 2). These C_6_ compounds derive from the lipoxygenase (LOX) catalyzed oxidation of linoleic or α-linolenic acids (Scheme 1) and were the main VOCs detected in all the tested olive oils. Different values of identified C_6_ aldehydes in the samples could be due to different acyl hydrolase activity and consequently good or poor availability of free polyunsaturated fatty acids. 3-Ethyloct-1,5-diene is derived from linoleic acid (Scheme 1). *Krvavica* oil was characterized by the highest level of hexanal, *Leccino* oil by (*E*)-hex-2-enal and (*Z*)-hex-3-enal was predominant in *Mašnjača* oil. According to Angerosa *et al.* [4], the level of each C6 compound depends on cultivar, which is confirmed by our results. 

**Table 2:** VOCs from *Krvavica*, *Mašnjača* and *Leccino* VOOs detected by HS-SPME/GC-MS.

No.	Compounds	RI	*Mašnjača*			*Krvavica*			*Leccino*		
			Area [%]			Area [%]			Area [%]		
			Min	Max	Mean ± SD	Min	Max	Mean ± SD	Min	Max	Mean ± SD
1.	Isoprene	<900	3.3	3.6	3.5 ± 0.15	1.6	1.7	1.7 ± 0.05	1.1	1.2	1.2 ± 0.05
2.	Ethyl acetate	<900	1.1	1.2	1.2 ± 0.50	0.8	0.8	0.8 ± 0.00	-	-	-
3.	Pent-1-en-3-one	<900	5.6	6.2	5.9 ± 0.30	2.0	2.0	2.0 ± 0.00	4.2	4.5	4.4 ± 0.15
4.	Pentan-3-one	<900	5.2	6.1	5.7 ± 0.45	3.8	3.9	3.9 ± 0.05	-	-	-
5.	(*E*)-Pent-2-enal	<900	0.6	0.6	0.6 ± 0.00	-	-	-	0.3	0.3	0.3 ± 0.00
6.	(*Z*)-Hex-3-enal	<900	21.9	25.0	23.5 ± 1.50	15.0	15.0	15.0 ± 0.00	-	-	-
7.	Hexanal	<900	-	-	-	8.8	9.4	9.1 ± 0.30	1.1	1.1	1.1 ± 0.00
8.	(*E*)-Hex-2-enal	<900	27.6	28.9	28.3 ± 0.65	41.6	44.4	43.0 ± 1.40	73.4	74.0	73.7 ± 0.30
9.	3-Ethyloct-1,5-diene(isomer I)	939	13.8	14.4	14.1 ± 0.30	3.3	4.2	3.8 ± 0.45	7.1	7.5	7.3 ± 0.20
10.	3-Ethyloct-1,5-diene(isomer II)	997	10.7	10.9	10.8 ± 0.10	3.3	4.1	3.7 ± 0.40	5.1	6.0	5.6 ± 0.45
11.	*trans*-β-Ocimene	1054	0.6	0.7	0.7 ± 0.05	1.9	2.5	2.2 ± 0.30	-	-	-
12.	α-Copaene	1380	1.6	2.2	1.9 ± 0.30	0.8	1.1	1.0 ± 0.15	0.0	0.1	0.1 ± 0.05
**Total identified**			(92.0%–99.8%)			(82.9%–89.1%)			(92.3%–94.7%)		

RI = retention indices on HP-5MS column; Min. = minimal percentage; Max. = maximal percentage; mean. = average percentage; SD. = standard deviation.

The level of C_5_ compounds (such as pent-1-en-3-one, pentan-3-one and (*E*)-pent-2-enal) was found to be lower in comparison to the C6 compounds. The percentage of pent-1-en-3-one that is positively correlated with bitter taste of VOO [7], in *Krvavica* oil was approximately two times lower than in *Leccino* oil and three times lower than in *Mašnjača* oil (Table 2). Additionally, in contrast to *Mašnjača* oil, *Krvavica* oil did not contain (*E*)-pent-2-enal and *Leccino* oil did not contain pentan-3-one. Several hydrocarbons such as α-copaene, *trans*-β-ocimene and two isomers of 3-ethyloct-1,5-diene, were detected in the investigated olive oils. *trans*-β-Ocimene was identified only in *Mašnjača* and *Krvavica* oils, whereas, α-copaene and 3-ethyloct-1,5-diene were detected in all the oils.

The composition of volatile compounds and sensory properties of monovarietal olive oils have been extensively studied [11,20]. Luna *et al.* [5] observed the differences in the composition of volatile compounds of olive oil obtained from 39 varieties. According to Angerosa *et al.* [21] accumulation of volatiles depends mostly on the variety since climatic and environmental conditions have an indirect effect e.g., by changing the degree of maturity of fruit trees. Since the investigated varieties were grown in the same orchard, and the fruits harvested in the same maturity index and processed in the same way, differences in the VOCs composition can be attributed to varietal characteristics.

Angerosa *et al.* [4] found that the concentration of (*E*)*-*hex-2-enal can be useful for distinguishing different oil varieties. Still, there are only a few studies investigating the volatile compounds of varietal VOOs from Croatia. Koprivnjak *et al.* [22] compared the volatiles of olive oils from two Istrian olive varieties (*Buza* and *Istarska bjelica*) and one introduced cultivar (*Leccino*). Varietal specificity of these oils has been established by the presence of high-value of the C_6_ aldehydes in cultivar *Leccino* and the C_6_ esters in cultivar *Buza*. In addition, *Leccino* oil from Istria contained the highest content of (*E*)-hex-2-enal [23], similar to our results. Brkić Bubola *et al.* [23] investigated VOCs and sensory characteristics of the oils from three Istrian olive varieties (*Buza*, *Črna* and *Rosinjola*) and found that the aroma compounds accumulate differently, depending on the varieties which indicate a close relation with the activity of an enzyme that is genetically determined. It can be helpful to understand the potential of indigenous varieties to produce high-quality monovarietal VOOs with specific organoleptic characteristics. The obtained results provide new data on the volatile profiles of oils obtained from two other Croatian cultivars *Mašnjača* and *Krvavica* which may be useful to distinguish between other Croatian as well as introduced cultivars.

### 2.3. Tocopherols, Chlorophylls and Carotenoids

Tocopherols, chlorophylls and carotenoids content of the studied oils are shown in Table 3. All the examined compounds were most abundant in *Leccino* oil, while in *Mašnjača* and *Krvavica* VOOs their content was lower. Variations in the content of these compounds is most probably influenced by variety, considering that olive fruits are from the same growing area, picked at the same ripening index and processed at the same conditions. These variations are in accordance with previous reports of other authors [24]. Research on the occurrence and levels of tocopherols in VOOs has shown that α-tocopherol comprises about 90% of total tocopherols content and the amount of α-tocopherol in the oil depends on the cultivar potential and technological factors [25]. According to some authors, there is a wide range of tocopherols content in VOOs. For example the quantity of tocopherols in Italian and Spanish oils has been variously reported to be 147–187 mg/kg [26]; 160–253 mg/kg [27]; 55–234 mg/kg [28]. Compared to them, Greek oils reached levels of α-tocopherol that are among the highest reported (127–370 mg/kg in 1994–1995; 98–333 mg/kg in 1995–1996 and 100–365 mg/kg in the 1996–1997 crop seasons) [29]. The levels of α-tocopherol may be related to the high levels of chlorophyll pigments and the concomitant requirement for singlet oxygen deactivation. Low amounts of α-tocopherol homologues β-tocopherol (~10 mg/kg), δ-tocopherol (~10 mg/kg) and γ-tocopherol (~20 mg/kg) are usually reported [24].

**Table 3:** The values of total phenols, α-tocopherol, γ-tocopherol, total chlorophylls and carotenoids of *Mašnjača*, *Krvavica* and *Leccino* VOOs.

VOO Sample	α-Tocopherol (mg/kg)	γ-Tocopherol (mg/kg)	Chlorophylls (mg/kg)	Carotenoids (mg/kg)
*Krvavica*	176.4 ± 0.9	33.7 ± 0.1	4.6 ± 0.2	1.8 ± 0.1
*Mašnjača*	223.9 ± 0.4	36.1 ± 0.2	4.2 ± 0.2	2.1 ± 0.1
*Leccino*	312.4 ± 0.3	42.5 ± 0.1	7.3 ± 0.6	4.2 ± 0.1

All values are expressed as mean of triplicate determinations ± SD.

The amounts of carotenoids in the investigated olive oils were relatively low, especially for those deriving from Croatian cultivars *Krvavica* and *Mašnjača* that contained about two times less than that obtained from *Leccino* oil. The fraction of β-carotene and other carotenoids in VOOs ranges from 0.5 to 15.0 mg/kg and depends on the variety of olive, degree of ripeness of the fruit, storage conditions, as well as the oil extraction method [30].

Similarly, chlorophylls content in all the samples was low and was higher in *Leccino* oil than in *Krvavica* and *Mašnjača* oils*.* The quantity of total chlorophylls in VOOs ranged from 5 to 35 mg/kg [30]. Like for other mentioned components, the quantity and composition of chlorophylls depend on the variety, degree of ripeness and storage conditions of fruits as well as on the method of the oil extraction. Three-phase centrifugation system gives oils with higher quantities of chlorophyll pigments in comparison with two-phase system, while the lowest quantities of chlorophyll are found in the oils obtained by pressing [30] which is in accordance with our results.

### 2.4. Total Phenolic Content and Phenolic Compounds Determined by LC-DAD

The obtained results show differences in content of total phenols among tested oils (Table 4), which are probably related to the genetic factors since all the tested oils were obtained from the olive fruits cultivated in the same area, climate conditions and processing techniques. The highest value of total phenols was found in *Leccino* oil (246.6 mg/kg) and in *Krvavica* and *Mašnjača* oils it was about two times lower. These results are in agreement with the findings of other authors [31,32]. For example, the mass fraction of total phenols in the oil samples from the olive fruit mixtures (Istria, Croatia) changed linearly in a strict correlation with fruit mass ratio of *Leccino* and *Istarska bjelica* cultivars [22]. The amount of phenolic compounds is an important factor in evaluation of the VOO quality because of their involvement in resistance to oxidation and the sharp bitter taste of the oil. The research conducted on olive oil chemical composition highlights that the polyphenols are remarkably variable according to the variety, the agronomic conditions, the state of ripeness and the technology of conservation [33]. According to Tsimidou [34], a wide range of total phenolic content has been reported (50–1,000 mg/kg), but values are usually between 100 and 300 mg/kg, which is similar to the values found in the investigated samples. Antioxidant activity measured with the DPPH antiradical assay also showed the highest value in hydrophilic fraction of *Leccino* oil (1.3 mmol TEAC/kg) and a statistically significant correlation was found between the total phenolic content and the DPPH values (r^2^ = 0.9856, *p* < 0.01).

**Table 4:** Total phenolic content and antioxidant activities (DPPH assay) of hydrophilic fractions of *Mašnjača*, *Krvavica* and *Leccino* VOOs.

VOO sample	Total phenols (mg GAE/kg)	DPPH (mmol TEAC/kg)
*Krvavica*	121.4 ± 1.9	0.5 ± 0.0
*Mašnjača*	143.4 ± 4.9	0.5 ± 0.0
*Leccino*	246.6 ± 2.6	1.3 ± 0.0

All values are expressed as mean of triplicate determinations ± SD.

LC coupled to DAD was utilized to characterize the polar compounds in *Mašnjača*, *Krvavica* and *Leccino* VOOs. The phenolic fraction chromatograms are quite similar for three samples, and Table 5 reports only positively detected phenolic compounds. *Leccino* and *Mašnjača* VOOs are characterized by high amount of 3,4-DHPEA-EDA, the dialdehydic form of decarboxymethylelenolic acid linked to hydroxytyrosol (also known as decarboxymethyl oleuropein aglycon). *Krvavica* VOO contains higher amounts of hydroxytyrosol and tyrosol than *Mašnjača* and *Leccino* VOOs and it is the only oil where vanillic acid and *p*-coumaric acid were detected. These results are similar to those reported by several authors for other monovarietal VOOs [12,32].

**Table 5:** Phenolic compounds from *Mašnjača*, *Krvavica* and *Leccino* VOOs hydrofilic fraction.

No.	Compounds	RT	LOD	LOQ	*Mašnjača*			*Krvavica*			*Leccino*		
			(mg/kg)	(mg/kg)	(mg/kg)			(mg/kg)			(mg/kg)		
					Min	Max	Mean ± SD	Min	Max	Mean ± SD	Min	Max	Mean ± SD
1.	Hydroxytyrosol ^a^	10.9	0.34	1.02	3.9	5.0	4.4 ± 0.8	14.9	21.9	18.4 ± 5.0	4.3	6.8	5.5 ± 1.8
2.	Tyrosol ^a^	13.4	0.40	1.21	3.9	4.9	4.4 ± 0.7	18.9	24.7	21.8 ± 4.1	3.0	4.8	3.9 ± 1.3
3.	Vanillic acid ^a^	15.1	0.04	0.11			nd	0.1	0.4	0.3 ± 0.2			nd
4.	*p*-Coumaric acid ^a^	19.2	0.23	0.70			nd	1.2	1.3	1.3 ± 0.1			nd
5.	3,4-DHPEA-EDA ^b^	22.4	0.94	2.86	60.2	64.2	62.2 ± 2.8	20.5	29.2	24.8 ± 6.2	146.5	159.7	153.1 ± 9.3
6.	Pinoresinol ^a^	25.2	0.16	0.47	0.5	2.1	1.3 ± 1.1	2.1	2.4	2.3 ± 0.2	2.7	3.2	3.0 ± 0.4
7.	Luteolin ^a^	25.7	0.46	1.40	4.8	5.1	4.9 ± 0.2	8.6	8.7	8.6 ± 0.0	5.0	6.2	5.6 ± 0.9
8.	*p*-HPEA-EDA ^b^	26.3	0.94	2.86	45.2	46.0	45.6 ± 0.6	40.2	45.6	42.9 ± 3.9	124.1	141.7	132.9 ± 12.5
9.	Apigenin ^a^	28.2	0.36	1.10	2.1	2.5	2.3 ± 0.3	2.7	2.9	2.8 ± 0.2	2.1	2.6	2.3 ± 0.3
10.	3,4-HPEA-EA ^b^	28.5	0.94	2.86	60.9	85.4	73.2 ± 17.3	73.0	95.5	84.2 ± 15.9	110.6	138.2	124.4 ± 19.5

rt = retention time; Min. = minimal value; Max. = maximal value; mean. = average value; LOD = limit of detection; LOQ = limit of quantification; SD. = standard deviation; nd = not detected (<LOD); ^a^ Identification based on RT and UV-Vis spectra of pure compounds; ^b^ Tentative identification by UV-Vis spectra and comparison of retention times with the literature data, and compounds dosed using the oleuropein calibration curve; 3,4-DHPEA-EDA, dialdehydic form of decarboxymethyl elenolic acid linked to hydroxytyrosol (decarboxymethyl oleuropein aglycon); *p*-HPEA-EDA, dialdehydic form of decarboxymethyl elenolic acid linked to tyrosol (decarboxymethyl ligstroside aglycon); 3,4-DHPEA-EA, oleuropein aglycon.

### 2.5. Antioxidant Activity of the Hydrophilic Fraction of the Olive Oils (DPPH Assay)

The model of scavenging stable radical DPPH is a widely used method to evaluate antioxidant capacities of natural products, and it has been used for olive oil [13]. In the present work, the antioxidant activity of polar extracts of three VOOs was evaluated. The antioxidant activity (DPPH assay) of tested VOO methanol-soluble extracts (hydrophilic fractions, HFs) was expressed in mmol TEAC/kg and presented in Table 4. The values range between 0.5 and 1.3 mmol TEAC/kg. Among the tested oils *Leccino* oil showed the highest value (1.3 mmol TEAC/kg), while two other oils exhibited two-fold lower value (0.5 mmol TEAC/kg). This difference in antioxidant activity of tested oils may depend on the total phenol content in the varieties. Most of the previous studies reported strong correlation between total phenols and antioxidant capacity [35]. Elevated free-radical scavenging activity for *Leccino* VOO was correlated to its high total phenolic content. According to Visioli *et al.* [36], phenolic compounds have strong antioxidant and free radical scavenging ability. Chain-breaking antioxidants, such as phenolic compounds, react with lipid radicals to form nonreactive radicals, interrupting the propagation chain. In fact, these compounds are able to donate an electron or hydrogen atom to the lipid radical formed during the propagation phase of lipid oxidation and stabilize the resulting phenoxyl radical by delocalizing the unpaired electron [14].

## 3. Experimental

### 3.1. Olive Oil Samples and Preparation of Their Hydrophilic Fractions

All tested oils were obtained from olive fruits from the orchards in north Dalmatian growing area (Zadar hinterland, Croatia) in 2012 where the olive trees are cultivated under identical agronomic and agrotechnical conditions. The olive trees (12-year-old) of the local and Italian varieties were from the same orchard (about 2 ha) and were planted in squares with 7 × 5 m spacing. The total number of olive trees in this orchard was 572. There was no irrigation. Three batches of oil fruits (replicates; each contained 200 kg of olive fruits) were collected from each variety at the same maturity index (MI = 4.3) and under the same agroclimatic conditions. Maturity index was calculated as a subjective evaluation of the skin colour and flesh as proposed by Uceda and Frias [37]. Only healthy olive fruits were handpicked, transported and stored under proper sanitary conditions. The olive fruits of each variety were processed separately in the oil extraction plant Molinova TG (Gruppo Pieralisi, Pieralisi S.p.A. Jesi, Italy) within 24 h of harvesting. The fruits were crushed with a hammer crusher and olive paste was malaxed at 26 ± 1 °C, for 35 min, in a olive paste mixer. The olive oil was separated by centrifugation through two phase decanter, without addition of warm water. Before and after the preparation of each olive oil sample, the extraction plant was cleaned. All samples were filtered through 25 mm GD/X 0.45 μm cellulose acetate filters (Whatman, Milan, Italy). After filtration, the olive oils were stored in dark glass bottles at 4 °C until the analyses.

Hydrophilic fractions (HF) of the oils were prepared by weighing 3 g of the oil and adding 5 mL of methanol–water (80:20 v/v) mixture in 20 mL screw cap test-tube. The mixture was blended in a ultrasonic bath apparatus for 15 min at 30 °C, and the emulsion was allowed to separate. The hydrophilic layer was placed in a round flask. The oil extraction was repeated another two times, the hydrophilic extracts were combined, and then evaporated under vacuum on a rotary evaporator at 30 °C. The residue was dissolved up to a final volume of 5 mL with the 80:20 methanol–water solution and filtered through a Whatman 13 mm GD/X 0.2 μm cellulose acetate syringe filter.

### 3.2. Determination of Oil Quality Parameters

Free acidity, expressed as % of oleic acid (%18:1); peroxide value, given as milliequivalents of active oxygen per kilogram of the oil (mEq O_2_/kg); and UV absorption characteristics (K_232_ and K_270_) were determined according to the analytical methods described in the European Union Commission Regulations EC 1989/2003 [19]. Free acidity, given as % of oleic acid, was determined by titration of the solution of oil dissolved in ethanol/diethyl ether (1:1) with ethanolic potash. Peroxide value, expressed in milliequivalents of active oxygen per kilogram of oil (mEq O_2_/kg), was determined as follows: a mixture of oil and chloroform/acetic acid was left to react with a solution of potassium iodide in darkness; the free iodine was then titrated with a sodium thiosulfate solution. K_232_ and K_270_ extinction coefficients were calculated from the absorption at 232 and 270 nm, respectively, with an UV spectrophotometer (Specord 200, Analytik Jena AG, Jena, Germany), using a 1% solution of the oil in cyclohexane and a path length of 1 cm.

### 3.3. Headspace Solid-Phase Microextraction (HS-SPME)

HS-SPME was carried out using a SPME fibre coated with layer of divinylbenzene/carboxen/ polydimethylsiloxane (DVB/CAR/PDMS) obtained from Supelco Co (Bellefonte, PA, USA). Before use, the fibre was conditioned according to the manufacturer instructions. For HS-SPME extraction 5 g of VOO was placed in a 15 mL glass vial and hermetically sealed with PTFE/silicone septa. The vial was maintained in a water bath at 40 °C during equilibration (15 min) and extraction (40 min) and was partially submerged so that the liquid phase of the sample was below the water level. All the experiments were performed under constant stirring (1000 rpm) with a magnetic stirrer. After sampling, the SPME fiber was withdrawn into the needle, removed from the vial, and inserted into the injector (250 °C) of the GC-MS for 6 min where the extracted volatiles were thermally desorbed directly to the GC column.

### 3.4. Gas Chromatography and Mass Spectrometry (GC-MS)

An Agilent Technologies (Palo Alto, CA, USA) gas chromatograph model 7890A equipped with mass selective detector, model 5975C and capillary column HP-5MS (5%-phenyl)-methylpolysiloxane Agilent J & W GC column, 30 m, 0.25 mm i.d., coating thickness 0.25 μm) was used. The flow rate of the helium carrier gas was 1.5 mL/min. The injector was operated in split mode (2:1 split ratio) at 260 °C. The column was maintained at 40 °C for 3 min, heated to 100 °C at a rate of 5 °C/min, heated to 260 °C at a rate of 3 °C/min and held to 260 °C for 3 min. MS conditions were as follows: source temperature 230 °C; quadrupole temperature 150 °C; transfer line temperature 270 °C; acquisition mode electron impact (EI 70 eV) by 3 scans s^−1^ and mass range *m/z* 29–350. The analyses were carried out in duplicate. The individual peaks were identified by comparison of their retention indices (relative to C_9_-C_25_ *n*-alkanes for HP-5MS) to those of authentic samples and literature as well as by comparing their mass spectra with the Wiley 275 MS library (Wiley, New York, NY, USA) and NIST98 (Gaithersburg, MD, USA) mass spectral database. The percentage composition of the samples was computed from the GC peak areas using the normalization method (without correction factors). The component percentages (Table 2) were calculated as mean values from duplicate GC-MS analyses.

### 3.5. Liquid Chromatography and Diode Array Detector (LC-DAD)

Detection and quantitative analyses of HF phenolic compounds were carried out using a LC-DAD method as described by Tuberoso *et al.* [38]. An ProStar HPLC system (Varian Inc., Walnut Creek, CA, USA) was employed, fitted with a pump module 230, an autosampler module 410, and a ThermoSeparation diode array detector SpectroSystem UV 6000lp (Thermo Separation, San Jose, CA, USA). Separation was obtained with a Gemini C18 column (150 × 4.60 mm, 3 μm, Phenomenex, Casalecchio di Reno, BO, Italy) using 0.2 M H_3_PO_4_ (solvent A), and acetonitrile (solvent B) at a constant flow rate of 1.0 mL/min, mixed in linear gradients as follows: t = 0 A:B (85:15, v/v), reaching 60:40 (v/v) in 30 min, then 40:60 (v/v) in 10 min, and finally at 100% B until 50 min. Before each injection the LC system was stabilized for 10 min with the initial A/B ratio (85:15, v/v). The injection volume was 10 μL. According to the optimal detection wavelength, the phenols analysis was performed at: 280 nm (hydroxytyrosol, tyrosol, vanillic acid, oleuropein, and ligstroside derivatives), 313 nm (*p*-coumaric acid), and 360 nm (luteolin and apigenin). Oleuropein and ligstroside derivatives were tentatively identified by comparison with literature data [39,40]. Chromatograms and spectra were elaborated with a ChromQuest V. 2.51 data system (ThermoQuest, Rodano, Milan, Italy). Stock standard solutions were prepared in methanol and working solutions in methanol–water (80:20 v/v). The method was validated in agreement with the International Conference on Harmonisation of Technical Requirements for Registration of Pharmaceuticals for Human Use (ICH) guidance note which describes validation of analytical methods [41] by determining linearity, limits of detection (LOD), limits of quantification (LOQ). The LODs and LOQs were used to establish the sensitivity of the method and were determined according to the equation LOD = 3.3σ/S and LOQ = 10σ/S, respectively (where σ = standard deviation of the blank, and S = slope of the calibration curve). The linearity was evaluated by preparing a standard mixture at six different concentrations, and analysing them by LC-DAD. The analyte peak areas were plotted against the corresponding concentrations, and the calibration curves were constructed by means of the least-squares method. All compounds were dosed using the calibration curve built with the respective standard, except oleuropein and ligstroside derivatives that were dosed using the oleuropein calibration curve. The correlation values were comprised between 0.9993 and 0.9999.

### 3.6. Determination of Tocopherols (Vitamin E)

A Shimadzu LC System (Shimadzu, Milan, Italy) equipped with a SCL-10A VP system control, a LC-10AD VP binary pump, a SIL-10AD VP autoinjector, connected to a Jasco 821-FP spectrofluorometer detector (Jasco Europe, Cremella, LC, Italy) was used. The operating conditions of detector were λ_ex_ = 298 nm and λ_em _= 325 nm. Injection volume was 20 μL. Separation was obtained with a Gemini C18 column (150 × 4.6 mm, 3 μm; Phenomenex) using acetonitrile–methanol (90:10, v/v) as eluent mixture at a flow rate of 0.8 mL/min. Five milligrams of the oil were weighed in a 1.8 mL vial, and added with 200 μL of chloroform and 790 μL of a mixture acetonitrile–methanol (50:50, v/v) and homogenized with a vibration mixer. α- and γ-tocopherols standard solutions were prepared in acetone, while working solutions were prepared to appropriate dilution with the eluent mobile phase. Linearity in the range 0.1–6 mg/kg was 0.9998.

### 3.7. Determination of Total Chlorophylls and Carotenoids

Solutions of 5% of the oil in acetone were prepared and absorbances at two different wavelengths (464 nm for carotenoids and 669 nm for chlorophylls) were measured with a Varian Cary 50 UV-visible spectrophotometer. Chlorophyll a and β-carotene stock standard solutions were prepared in acetone, as well as working solutions, that were prepared with proper dilutions (0.1–2.0 mg/kg, r = 0.9997 and 0.02–0.50 mg/kg, r = 0.9994 for chlorophyll a and β-carotene, respectively).

### 3.8. Determination of Total Phenolic Content (Folin-Ciocalteu Assay)

Total phenolic content of the HF was estimated spectrophotometrically with a modified Folin-Ciocalteu method [42]. Briefly, 100 μL of HF was added to 500 μL of Folin-Ciocalteu phenol reagent. After 5 min, 3 mL of 10% Na_2_CO_3_ (w/v) was added, the mixture was shaken, and then diluted with water to a final volume of 10 mL. After a 90 min incubation period at room temperature, the absorbance was read at 725 nm on a 10 mm quartz cuvette using a Varian Cary 50 spectrophotometer, against a blank. The total polyphenol content results, expressed as mg/kg of gallic acid equivalent (GAE), were obtained using a calibration curve of a freshly prepared gallic acid standard solution (5–100 mg/kg, r = 0.9999).

### 3.9. Free Radical Scavenging Activity (DPPH Assay)

The *in vitro* antiradical activity of the HF was determined by spectrophotometric analysis that used DPPH and the data were expressed as Trolox equivalent antioxidant capacity (TEAC) [42]. This assay is based on the ability of the antioxidant to scavenge the radical cation 1,1-diphenyl-2-picrylhydrazyl radical (DPPH). Fifty microlitres of HF sample was dissolved in 2 mL of 0.04 mmol/L DPPH in methanol. Spectrophotometric readings were carried out with a Varian Cary 50 spectrophotometer at 517 nm, using a 10 mm plastic cuvette after an incubation period of 60 min in dark and at room temperature. A Trolox calibration curve in the range 0.02–1.00 mM was prepared (r = 0.9997), and data were expressed in Trolox equivalent antioxidant capacity (TEAC, mmol/kg).

## 4. Conclusions

All the quality parameters of the investigated olive oils were within the limits established for the extra virgin olive oil category. Characterization of *Mašnjača* and *Krvavica* oils in comparison to *Leccino* oil showed significant differences among various parameters. The olive oil from the Italian *Leccino* cultivar contained up to two times higher amounts of tocopherols, chlorophylls, carotenoids and total phenols in comparison with Croatian *Mašnjača* and *Krvavica* oils*.* Its HF showed also much higher antioxidant activity in DPPH test which is related to higher content of phenolics. The results show that the Croatian autochthonous cultivars *Mašnjača* and *Krvavica* give oil that contain moderate amounts of tocopherols and phenolics. The results of volatile profile investigation show that the headspace of both *Mašnjača* and *Krvavica* oils represent more complex flavour in comparison with *Leccino* oil which does not contain ethyl acetate, pentan-3-one, (*Z*)-hex-3-enal and *trans*-β-ocimene. Additionally, *Krvavica* oil could be distinguished from *Mašnjača* oil by the lack of (*E*)-pent-2-enal, the presence of hexenal and higher ratio of (*E*)-hex-3-enal to (*Z*)-hex-3-enal*.* Headspace volatile profiles show differences in composition that suggest differences in perceptible aroma and could be useful to distinguish olive oils from different cultivars. In summary, *Mašnjača* and *Krvavica* oils have moderate amounts of tocopherols and phenolics, but characteristic headspace profiles that could be welcome in a Mediterranean diet, particularly regarding sensory attributes caused by a more complex headspace profile, but more detailed research is necessary, including panel test sensory analysis. In addition, further analyses, such as fatty acids and sterols composition are necessary for more complete evaluation of these two autochthonous cultivars.
